# Use of Commercially Produced Medical Education Videos in a Cardiovascular Curriculum: Multiple Cohort Study

**DOI:** 10.2196/27441

**Published:** 2021-10-07

**Authors:** Sean Tackett, David Green, Michael Dyal, Erin O'Keefe, Tanya Emmanuelle Thomas, Tiffany Nguyen, Duyen Vo, Mausam Patel, Christopher J Murdock, Erin M Wolfe, Lina A Shehadeh

**Affiliations:** 1 Division of General Internal Medicine Johns Hopkins Bayview Medical Center Baltimore, MD United States; 2 Department of Medical Education University of Miami Miller School of Medicine Miami, FL United States; 3 Department of Veteran Affairs Miami, FL United States; 4 Division of Cardiology Department of Medicine University of Miami Miller School of Medicine Miami, FL United States; 5 Interdisciplinary Stem Cell Institute University of Miami Miller School of Medicine Miami, FL United States

**Keywords:** commercial videos, flipped classroom, organ-systems courses, medical education, medical students, teaching, education, health science education, e-Learning

## Abstract

**Background:**

Short instructional videos can make learning more efficient through the application of multimedia principles, and video animations can illustrate the complex concepts and dynamic processes that are common in health sciences education. Commercially produced videos are commonly used by medical students but are rarely integrated into curricula.

**Objective:**

Our goal was to examine student engagement with medical education videos incorporated into a preclinical Cardiovascular Systems course.

**Methods:**

Students who took the first-year 8-week Cardiovascular Systems course in 2019 and 2020 were included in the study. Videos from Osmosis were recommended to be watched before live sessions throughout the course. Video use was monitored through dashboards, and course credit was given for watching videos. All students were emailed electronic surveys after the final exam asking about the course’s blended learning experience and use of videos. Osmosis usage data for number of video views, multiple choice questions, and flashcards were extracted from Osmosis dashboards.

**Results:**

Overall, 232/359 (64.6%) students completed surveys, with rates by class of 81/154 (52.6%) for MD Class of 2022, 39/50 (78%) for MD/MPH Class of 2022, and 112/155 (72.3%) for MD Class of 2023. Osmosis dashboard data were available for all 359 students. All students received the full credit offered for Osmosis engagement, and learning analytics demonstrated regular usage of videos and other digital platform features. Survey responses indicated that most students found Osmosis videos to be helpful for learning (204/232, 87.9%; *P*=.001) and preferred Osmosis videos to the traditional lecture format (134/232, 57.8%; *P*<.001).

**Conclusions:**

Commercial medical education videos may enhance curriculum with low faculty effort and improve students’ learning experiences. Findings from our experience at one medical school can guide the effective use of supplemental digital resources for learning, and related evaluation and research.

## Introduction

Short instructional videos can make learning more efficient through the application of multimedia principles [1], and video animations in particular can illustrate the complex concepts and dynamic processes that are common in health sciences education [2]. Medical students usually select and use videos in a self-directed manner; however, videos are increasingly being incorporated into the formal curriculum to enhance and reinforce knowledge, similar to the use of textbook or journal readings to supplement faculty-developed resources in traditional medical school curricula.

Published descriptions of instructional videos for medical students focus on faculty-created videos [3-6], and published advice for creating instructional videos [7,8] could imply that video development should be added to the list of skills that faculty should learn. However, optimizing the educational quality of instructional videos requires careful planning, familiarity with technology, and drawing ability [9]. Faculty may not have the time or interest to learn these skills, and institutions may not have adequate resources to support them. Working with companies that have clearly defined processes and the infrastructure for educational video production is one option for developing high-quality videos efficiently; however, such an option may be most appropriate for faculty who view video creation as a form of scholarship and when videos can be distributed broadly [10].

Purchasing videos produced by health education companies may be more practical than either asking faculty to create videos independently or to cocreate them with education companies. Although digital educational resources to supplement learning are universally purchased by students and invested in by a growing numbers of institutions, we are unaware of reports describing their implementation and evaluation as part of formal curricula. Therefore, our goal was to evaluate the incorporation of videos on a digital platform in a preclerkship curriculum.

## Methods

This was a three-cohort study that examined student engagement with medical education videos in the Cardiovascular Systems course for first-year medical students at the University of Miami Miller School of Medicine (Miami, Florida). The videos selected were from Osmosis [11], a digital education platform that includes videos, flashcards, and case-based multiple-choice questions. The University of Miami is a private institution and the curriculum has been in place for over 20 years. The Cardiovascular Systems course lasts 8 weeks and runs twice each spring. The cohort of MD students completes the course first (usually in January-March) and MD/MPH students complete the course second (usually in April-May). The MD/MPH cohort has a model that is structured around problem-based learning (PBL) time. The students attend in-class lectures (as in the MD program), meet twice a week in small groups of 10 students to analyze cases, and then the full class meets once a week in a PBL wrap-up session. However, the content and number of lectures are equal between the MD and MD/MPH classes. We included MD and MD/MPH students from the class of 2022 who took the course in 2019 and MD students from the class of 2023 who took the course in 2020. For each of the 3 cohorts, the study applied to the 8-week duration of the course. We did not include the MD/MPH students who took the course in 2020 because their learning experiences were altered by the COVID-19 pandemic. For example, the students had all of their classes and assessments virtually.

Osmosis was selected because its video topics were organized similar to the course topics, and thus the faculty felt that the videos would provide a conceptual foundation that better prepared students for live activities in the course and that the videos could be useful for review after the content was covered in the course.

Prior to 2019, course faculty had created 17 videos on cardiovascular system physiology, lipids, and coronary blood flow lectures to be used in the course, called “Cane Academy” videos. These videos were intended to replace lectures, and were longer (average duration of 15 minutes) and more detailed than typical Osmosis videos (average duration of 7 minutes). Cane Academy videos were developed for multiple courses at the University of Miami Miller School of Medicine [12-14]. Students continued to have access to Cane Academy videos during the 2019 and 2020 Cardiovascular Systems courses. Most of the course topics did not have Cane Academy videos available and relied on traditional classroom lectures; Osmosis videos were assigned for these topics. The comparison in this study is not of Osmosis vs Cane Academy videos but rather the addition of Osmosis to our curriculum as a supplemental resource.

All students were given free access to Osmosis Prime and were advised by instructors to watch 1-2 specific videos among 58 Osmosis cardiovascular videos before each classroom session. In both years, engagement with Osmosis accounted for 4% of the students’ course grade. The course director monitored students’ engagement on a weekly basis using Osmosis dashboards that showed video-viewing data, and each week, the course director emailed histograms of the video-viewing data to each student that showed individual views compared to views of the whole class. Credit was provided based on the course director’s estimate of students having completed 60% of expected video views and associated questions.

All students were emailed electronic surveys after the final exam asking about the course’s blended learning experience and use of videos, including items with Likert-scale response options and open-ended prompts. The surveys were open for 10 days. Likert-scale items asked respondents to “Indicate the degree to which you agree/disagree with the following statements.” Items with open-ended prompts asked for students to comment on what they liked and would change about the “blended learning experience” and did not specifically ask about Osmosis. Osmosis usage data for number of video views, multiple choice questions, and flashcards were extracted from Osmosis dashboards.

We calculated descriptive statistics for Likert-scale items and Osmosis usage data. We compared differences in the proportions of students who agreed or strongly agreed to a survey items across cohorts using the χ^2^ test. Based on visual inspection of histograms of cohort usage data, Osmosis usage did not fit a normal distribution. Accordingly, we report medians and IQRs as summary data, and used the Kruskal-Wallis test to examine differences across the 3 cohorts. We used Excel and Stata (StataCorp 2013) for statistical analyses. Responses to the open-ended survey questions were collated into a Microsoft Word document and analyzed independently by two individuals using an editing analysis method. Using thematic analysis [15], we report the themes that emerged from this analysis.

This study was carried out in accordance with the recommendations of the University of Miami Institutional Review Board (IRB protocol 2019-0323). All online survey results were deidentified, therefore negating the need for consent forms.

## Results

Overall, 232/359 (64.6%) of the students completed surveys, with rates by class of 81/154 (52.6%) for MD class of 2022, 39/50 (78%) for MD/MPH class of 2022, and 112/155 (72.3%) for MD class of 2023. Osmosis dashboard data were available for all 359 students.

Student surveys indicated that most students (134/232, 57.8%) preferred Osmosis videos to traditional lectures, with greater proportions of students in 2020 preferring Osmosis compared to those in 2019 (*P*<.001). Preference for Cane Academy videos did not vary by class (Table 1).

**Table 1:** Respondents who agreed or strongly agreed with each item on the postcourse survey.^a^

Survey item	All (N=232), n (%)	MD 2022 (n=81), n (%)	MD/MPH 2022 (n=39), n (%)	MD 2023 (n=112), n (%)	*P* value^b^
Osmosis videos were easy to access	230 (99.1)	80 (98.8)	39 (100.0)	111 (99.1)	.40
Osmosis video quality (audio, visuals, and other technical aspects) was acceptable	226 (97.4)	76 (93.8)	38 (97.4)	112 (100.0)	.01
The Osmosis videos were helpful to my learning	204 (87.9)	64 (79.0)	34 (87.2)	106 (94.6)	.001
The blended classroom (ie, watching the online Osmosis videos before coming to class) allowed me to reflect on a deeper level…more so than a traditional lecture-based course	197 (84.9)	63 (77.8)	33 (84.6)	101 (90.2)	.01
The Osmosis self-assessment questions were helpful to my learning	177 (76.3)	48 (59.3)	32 (82.1)	97 (86.6)	<.001
I prefer the Osmosis videos to traditional lectures (live or via Panopto recordings)	134 (57.8)	32 (39.5)	20 (51.3)	82 (73.2)	<.001
I prefer the Cane Academy videos to traditional lectures (live or via Panopto recordings)	127 (54.7)	43 (53.1)	18 (46.2)	66 (58.9)	.16

^a^MD 2022 and MD/MPH 2022 took the class in the spring of 2019 and MD 2023 took the class in the spring of 2020.

^b^*P* values are based on the χ^2^ test for proportions across the 3 classes.

Student comments to the open-ended prompts described appreciation for having multiple online resources suggested to supplement their learning (eg, “Access to numerous resources allowed me to understand the content from different perspectives…each resource that I used helped reinforce the other”) and greater control over their learning experience (eg, “I loved the autonomy of my schedule to really figure out what learning strategies work best for me”). Students mentioned Osmosis as particularly helpful in preparing for lecture or PBL sessions (eg, “The Osmosis videos did a great job introducing concepts before watching lectures. This method of ‘priming’ was helpful in formulating questions that I could then ask in small group sessions and overall more effectively retain class content”). Suggestions for improvement in Osmosis video implementation related to the course credit linked to Osmosis usage (eg, “The Osmosis videos shouldn’t be mandatory but rather leave recommended videos to supplement the material in class”).

All students received the full credit offered for Osmosis engagement. Across the 3 classes, on average per week, students watched a median of 9 videos, and completed a median of 74 multiple choice questions and a median of 80 flashcards. Video views were greater in 2020 than in 2019 (*P*<.001), but flashcard usage was lower (*P*<.001) (Table 2).

**Table 2:** Median (IQR) student usage of Osmosis during the 8-week Cardiovascular Systems course.

Usage metric			All (N=359)		MD 2022^a^ (n=154)	MD/MPH 2022^a^ (n=50)		MD 2023^a^ (n=155)
**Total for course**								
	Video views^b^	75 (56-103)		61 (50-73)		57 (42-69)	105 (89-136)	
	MCQs^c^ completed	593 (349-779)		595 (349-737)		552 (349-820)	593 (356-815)	
	FCs^d^ completed^b^	637 (10-900)		872 (787-1036)		854 (716-945)	2 (0-32)	
**Average per week**								
	Video views^b^	9 (7-13)		8 (6-9)		7 (5-9)	13 (11-17)	
	MCQs completed	74 (44-97)		74 (44-92)		69 (44-103)	74 (45-102)	
	FCs completed^b^	80 (1-113)		109 (98-130)		107 (90-118)	0 (0-4)	

^a^MD 2022 and MD/MPH 2022 took the class in the spring of 2019 and MD 2023 took the class in the spring of 2020.

^b^*P*<.001 based on the Kruskal-Wallis test for differences across the 3 classes.

^c^MCQ: multiple choice question.

^d^FC: flashcard.

**Figure 1:**
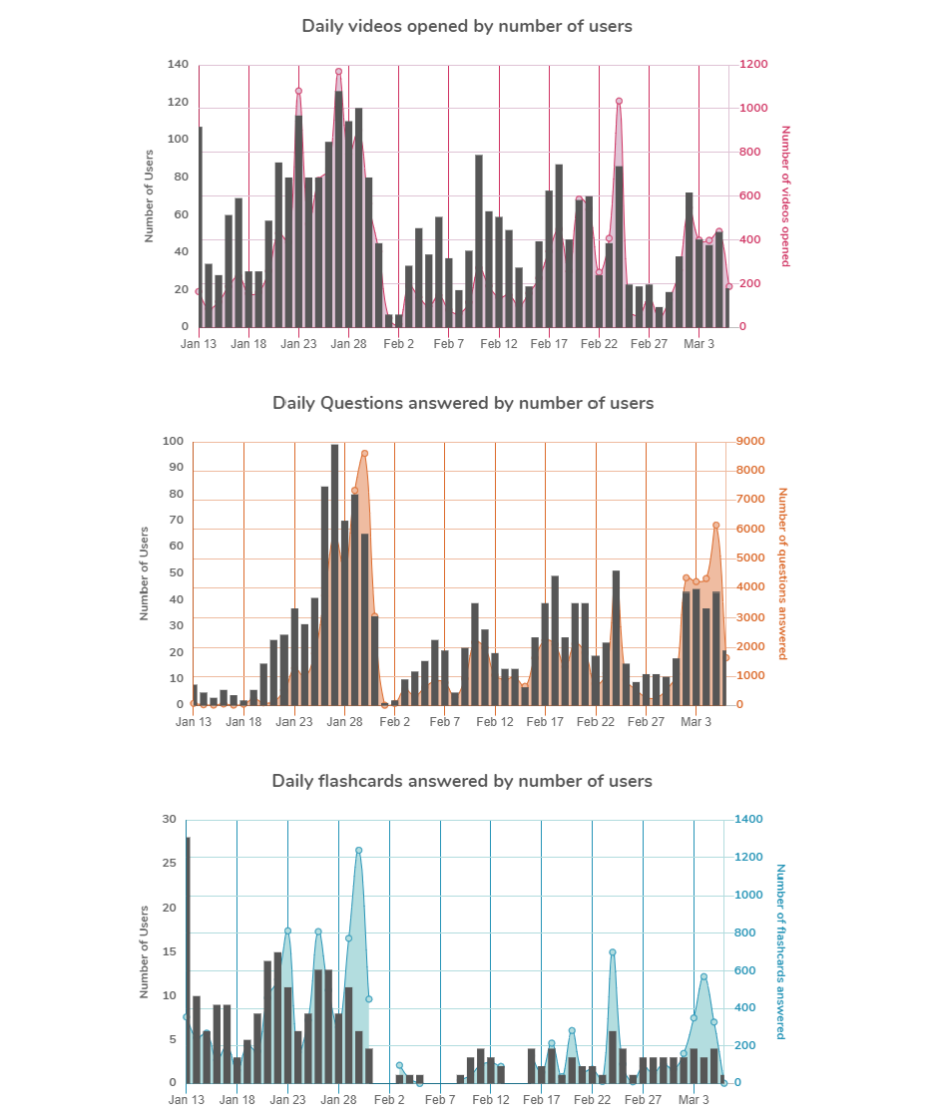
Osmosis dashboard data for MD class of 2023 showing video views, multiple choice questions, and flashcards for all users during the 8-week Cardiovascular Systems course.

Overall usage varied over time throughout the course (Figure 1). Usage of videos, multiple choice questions, and flash cards tracked together, likely indicating that students were more prone to use multiple platform features once they began using one feature.

## Discussion

This evaluation of Osmosis videos in a preclinical cardiovascular systems course at one medical school suggests that videos were acceptable to students, and Osmosis usage expanded beyond video viewing to include answering multiple choice questions and flashcards.

Commercially published resources in the form of textbooks and journal articles are taken for granted in health sciences education; however, it remains a challenge for medical educators to determine the best approaches to incorporate commercially produced digital learning resources for similar purposes. Implementing digital learning resources in medical education has been described as requiring the consideration of 3 technological factors (ie, relative advantage, ease of initial adoption, and availability), 4 teacher factors (ie, attitude toward change, capabilities, pedagogical beliefs and practice, and control), and 4 contextual factors (ie, bureaucracy, politics and purpose, prioritization of research, and culture and discipline) [16]. Technological factors were addressed in our course by selecting a product that students perceived as accessible, easy to use, and preferred over traditional approaches, as illustrated by student survey responses. Most teacher factors were in place as our core group of faculty, including course directors, were enthusiastic about innovation. Some participating faculty expressed resistance, but felt more comfortable after they were invited to edit Osmosis video scripts or point out areas in videos (eg, oversimplification) that were inconsistent with what they typically taught. We previously described a careful choreography that is needed when aligning face-to-face elements with technology-enhanced solutions (such as educational videos) [17]. Part of this blend is to ensure that measurable learning objectives are aligned with educational interventions, formative feedback, and summative assessments. Obtaining faculty feedback and buy-in throughout this blended learning implementation process enriched opportunities for successful course redesign efforts [18]. Faculty generally saved time by using a resource that aligned with their content. Finally, contextual factors favored innovation. The Dean of University of Miami Miller School of Medicine was promoting culture change and curriculum transformation. In obtaining leadership support, we explicitly aligned changes in the Cardiovascular Systems course to leadership goals.

Empiric reports of commercial digital resources for medical student learning are limited and usually focus on licensing exam preparation [19-26]. One report found highly variable usage and a smaller proportion of students using Osmosis when it was provided to students for free without linkage to the curriculum. Lack of faculty champions and time to learn how to use Osmosis were described as barriers to adoption [27]. Although we observed variation in how students used Osmosis in the Cardiovascular Systems course, they all engaged with the platform. We primarily attribute this high usage to providing course credit for Osmosis use, which incentivized students to overcome the barrier of finding time to adopt Osmosis as a new learning resource. Weekly feedback (from the course director to students) on student engagement, which required minimal time and effort, may have also incentivized students to engage with the platform. It is also possible that we helped students avoid the paradox of choice [28], in which having too many options creates distress and decreases the chance that a choice will be made. By offering and supporting a single platform aligned with the course, we simplified their choices, which may have allowed more cognitive effort to be available for learning.

Osmosis learning analytics were useful for tracking student behaviors and providing feedback that compared students to their peers. Learning analytics have examined learning behaviors for other medical education videos [29,30]; however, to our knowledge, this is the first report for videos integrated into a medical student curriculum. Analytics prompted interesting observations. For example, the relatively low usage of Osmosis flashcards was most likely because the students were already accustomed to using Anki flashcards. The peak engagement with the Osmosis platform was before the midterm and final exams, providing insights into how students used Osmosis to study (eg, reviewed videos and practiced questions more intensely before exams). Future work would be required to understand how learning analytics impacted students, as some have expressed concern that learner dashboards may adversely influence learning [31].

Important limitations must be kept in mind when interpreting this work. Although we captured a majority of students from each class with our course evaluation survey and our overall response rate was on par with previously published survey studies [32], our survey relied on student self-report and Likert-scale response options, which are subject to potential measurement error [33]. Although we included 3 classes of students over 2 years, rendering a reasonably large sample, our study was conducted at a single institution; thus, whether our findings would hold in other contexts would require further empiric investigation. We did not design the study to make head-to-head comparisons for Osmosis and Cane Academy videos, and we did not systematically evaluate the perceptions or activities of faculty in the course. Osmosis videos appeared to be comparable in many respects to Cane Academy videos; however, future work is required to understand if Osmosis may have offered a return on investment by decreasing faculty effort. Histograms of cohort usage data were inspected visually without formal statistical testing. We did not design our study to examine relationships between usage and knowledge gains; future work that potentially uses controlled study designs or assesses student behaviors outside of the Osmosis platform may help us to better understand how Osmosis might influence student learning.

In conclusion, commercial medical education videos may enhance curriculum with low faculty effort, improve students’ learning experiences, and offer a favorable return on investment to medical schools. This short communication can guide ideas for more robust evaluation and research to better understand how engagement with a digital educational platform and its analytics can influence learning.

## Abbreviations

PBL: problem-based learning
